# Postoperative Pain and Healthcare Utilization Following Chiari Malformation Decompression: A Multicenter Retrospective Cohort Study

**DOI:** 10.7759/cureus.109886

**Published:** 2026-05-29

**Authors:** Taylor G Kreul, Tyler D Krall, Susannah Cowley, Scotty Wynn, Mitchel Nelson, Daniel Wieland, Ty Duffy, Kristin Nosova, Mazen Zaher

**Affiliations:** 1 Plastic and Reconstructive Surgery, University of Arizona College of Medicine - Phoenix, Phoenix, USA; 2 Neurosurgery, University of Arizona College of Medicine - Phoenix, Phoenix, USA; 3 Anesthesia, University of Arizona College of Medicine - Phoenix, Phoenix, USA; 4 Neurological Surgery, Banner - University Medical Center Phoenix, Phoenix, USA

**Keywords:** chiari type 1 malformation, chiari type 2 malformation, enhanced recovery after surgery (eras) protocols, multimodal analgesia, postoperative pain relief

## Abstract

Introduction: Postoperative pain following Chiari malformation decompression is frequently severe and may contribute to substantial postoperative healthcare utilization. Although multimodal analgesic strategies are increasingly utilized across surgical specialties, postoperative pain management practices following Chiari decompression remain variable and poorly characterized. This exploratory retrospective study aimed to characterize postoperative analgesic practices and associated healthcare utilization outcomes following Chiari decompression.

Methods: A retrospective cohort study was conducted of adult patients undergoing Chiari decompression across four institutions between 2016 and 2023. Postoperative analgesic regimens, including opioid and non-opioid medications, were recorded. Outcomes included postoperative pain scores, length of stay (LOS), postoperative imaging utilization, emergency department (ED) visits within 60 days of surgery, and readmissions.

Results: Twenty-seven patients met inclusion criteria. Postoperative analgesic regimens were heterogeneous, with all patients receiving opioid medications and variable use of non-opioid adjuncts. Eleven patients (40.7%) presented to the ED during the postoperative period, most commonly for headache or pressure-related symptoms. These presentations frequently required additional pharmacologic management and diagnostic imaging, including computed tomography in 63.6% of cases and magnetic resonance imaging in 18.2%. Two patients were subsequently found to have cerebrospinal fluid leaks requiring operative intervention. Exploratory analysis demonstrated lower postoperative day 0 pain scores among patients receiving non-steroidal anti-inflammatory drugs (NSAIDs).

Conclusions: Postoperative pain and healthcare utilization following Chiari decompression remain important clinical challenges. In this exploratory retrospective cohort, postoperative analgesic practices were variable, and a substantial proportion of patients required postoperative ED evaluation and additional imaging. These findings highlight the persistent postoperative symptom burden experienced by this patient population and may help inform future prospective evaluation of perioperative pain management pathways and postoperative care strategies following Chiari decompression.

## Introduction

Effective postoperative pain management is essential to patient recovery, yet it remains a persistent challenge in many surgical populations. Opioids have traditionally served as the primary modality for postoperative analgesia but are associated with well-documented adverse effects, including nausea, constipation, sedation, respiratory depression, and risk of dependence [1,2]. In response, multimodal analgesia (MMA) strategies have been widely adopted, incorporating non-opioid medications such as acetaminophen, non-steroidal anti-inflammatory drugs (NSAIDs), and adjunctive agents to target multiple pain pathways while minimizing opioid exposure [3-5]. These approaches are central to Enhanced Recovery After Surgery (ERAS) protocols, which have been associated with improved patient outcomes, reduced complications, and decreased healthcare costs across a variety of surgical specialties [6,7].

Despite these advances, the application of ERAS principles in neurosurgery is inconsistent, with protocols primarily developed for elective spinal procedures [8-15]. While not widely adopted, ERAS in neurosurgery confers similar benefits as seen in other surgical fields [9-15]. Obstacles persist, such as the challenge of managing complex neurosurgical cases, the lack of standardized protocols, and the need for effective interdisciplinary teamwork [8-10]. Patients undergoing Chiari malformation decompression represent a unique and understudied population in this context, where postoperative pain is both common and difficult to manage [16-18]. Chiari I and II malformations are congenital disorders of hindbrain development in which the cerebellar tonsils or brainstem, respectively, herniate through the foramen magnum, often resulting in headaches, neck pain, and neurological deficits requiring surgical decompression [18,19]. Despite being relatively common indications for posterior fossa surgery in adults, postoperative recovery in Chiari patients remains uniquely challenging due to the intensity of occipital pain, high variability in symptom resolution, and frequent emergency department (ED) utilization for persistent headache or pressure symptoms [16,17,20,21].

Importantly, poorly controlled postoperative pain in this population extends beyond patient discomfort and may contribute to substantial healthcare utilization. These encounters frequently result in additional opioid administration, repeat imaging, and, in some cases, hospital readmission, representing a meaningful burden to both patients and healthcare systems. Despite this, there remains a lack of standardized, procedure-specific pain management protocols tailored to Chiari surgery, and current practices are highly variable across providers and institutions [22].

Given the absence of established pathways and the potential consequences of inadequate pain control, there is a need to better characterize current postoperative analgesic practices and their associated clinical outcomes in patients undergoing Chiari decompression. The aim of this exploratory retrospective multicenter study was to characterize postoperative analgesic practices and healthcare utilization following Chiari decompression in adult patients, with primary outcomes including postoperative ED visits and imaging utilization during the postoperative follow-up period. This study evaluates real-world pain management strategies across multiple institutions, with particular attention to patterns of healthcare utilization, including ED visits, imaging, and readmissions. By highlighting the burden of postoperative pain and variability in management, this work aims to inform the development of standardized, opioid-sparing protocols to improve outcomes in this challenging patient population.

## Materials and methods

This retrospective study evaluated patients who underwent surgical correction for Chiari malformation within four Level I trauma centers across Arizona. Institutional Review Board approval was obtained prior to data collection. Patient data were extracted from the Cerner electronic medical record system and entered into REDCap, a secure, HIPAA-compliant platform (Research Electronic Data Capture; Vanderbilt University, Nashville, Tennessee, USA) used for study data management.

Adult patients aged 18 years or older with a diagnosis of Chiari I or Chiari II malformation who underwent Chiari-related surgical intervention between 2016 and 2023 were eligible for inclusion. Patients were identified retrospectively through electronic medical record query using the International Classification of Diseases, Tenth Revision (ICD-10) diagnosis code Q07.00 for Arnold-Chiari syndrome without spina bifida. Surgical procedures encompassed posterior fossa decompression, suboccipital craniectomy, and decompression with C1 laminectomy. Patients were excluded if their Chiari-related surgical intervention occurred outside the IRB-approved study period, if they did not undergo Chiari-related surgery, or if operative or postoperative documentation was insufficient for outcomes assessment. A total of 382 patients were initially identified through electronic medical record query using ICD-10 diagnosis code Q07.00 (Arnold-Chiari syndrome without spina bifida). Patients were excluded if they did not undergo Chiari-related surgical intervention (n = 205), underwent surgery outside the IRB-approved study period (n = 112), or had insufficient operative or postoperative documentation for analysis (n = 38), resulting in a final study cohort of 27 patients.

Medical records of included patients were reviewed to characterize postoperative analgesic regimens. Analgesic variables included type and number of medications administered, opioid consumption measured in morphine milligram equivalents (MME), use of intravenous versus oral formulations, timing of transition from intravenous to oral analgesia, and use of non-opioid adjuncts such as NSAIDs, acetaminophen, butalbital, diazepam, methocarbamol, ketorolac, and ketamine. Postoperative pain scores were collected retrospectively from nursing and provider documentation within the electronic medical record. Pain was assessed using the institutionally documented numeric rating scale, typically ranging from 0 to 10. Pain scores were obtained retrospectively from routine nursing and provider documentation within the electronic medical record. Because assessments were performed as part of routine clinical care, the frequency and timing of pain score documentation varied between patients and institutions. All available pain scores documented during each postoperative day were collected and averaged to generate a daily postoperative pain score. Missing pain scores were not imputed. Because pain assessments were not standardized, patients receiving NSAIDs and those not receiving NSAIDs may not have had pain scores recorded at equivalent time points.

Clinical outcomes included hospital length of stay (LOS), number and type of postoperative imaging studies, ED visits, readmissions, and need for reoperation. Given the association between unplanned healthcare utilization and increased cost and resource burden, ED visits, readmissions, and imaging utilization were evaluated as markers of healthcare resource use. The primary outcomes were postoperative ED visits and imaging utilization. Postoperative ED visits and readmissions were assessed within 60 days of surgery. Postoperative ED visits were identified through review of the participating health systems’ electronic medical records, and out-of-network ED presentations may therefore have been underdetected.

For the purposes of descriptive categorization, MMA was defined as the use of more than one analgesic modality for postoperative pain control, particularly the combination of opioid and non-opioid medications targeting different pain pathways. Non-opioid adjuncts included medications such as acetaminophen, NSAIDs, butalbital-containing medications, muscle relaxants, benzodiazepines, ketorolac, and ketamine. Because this was a multicenter retrospective study, no standardized MMA protocol was utilized across participating institutions, and analgesic regimens reflected individual provider and institutional practice patterns.

Descriptive statistics were used to summarize demographic characteristics, postoperative analgesic regimens, pain scores, and healthcare utilization outcomes. Continuous variables were reported as means with ranges, and categorical variables were summarized as frequencies and percentages. Given the small sample size and heterogeneity of treatment strategies, statistical analysis was primarily exploratory. Comparative analysis was limited to evaluation of postoperative pain scores among patients who did and did not receive NSAIDs using the Wilcoxon rank-sum test. Statistical significance was defined as p < 0.05. Given the small sample size and heterogeneity of treatment strategies, statistical analysis was primarily exploratory and descriptive. Additionally, formal inter-rater reliability analysis was not performed.

## Results

A total of 27 patients were included in this study (21 females, six males), with a mean age of 36.1 (range, 20-77) years at the time of surgery. Most patients (n = 24; 88.9%) were diagnosed with Chiari I malformation. Surgical procedures included posterior fossa decompression, occipital craniectomy, and suboccipital decompression with C1 laminectomy. Patient demographics and operative characteristics are summarized in Table 1.

**Table 1:** Demographic Characteristics and Comorbidities. BMI: body mass index

Variable	Number of Patients (n)
Patients	27
Male	5
Female	22
Age, average (range)	36.1 (20-77)
BMI, average (range)	31.8 (21.6-53.6)
Chiari 1 malformation	24
Chiari 2 malformation	3
Current smoker	5
Former smoker	8
Never smoker	14
Migraine or headache disorder	23
Syrinx	9
Depression	6
Anxiety	5
Chronic pain syndrome	5
Hypertension	3
Syringomyelia	3
Opioid use disorder	2
Diabetes mellitus	1
Scoliosis	1
Prior cervical spine fusion	0

**Figure 1:**
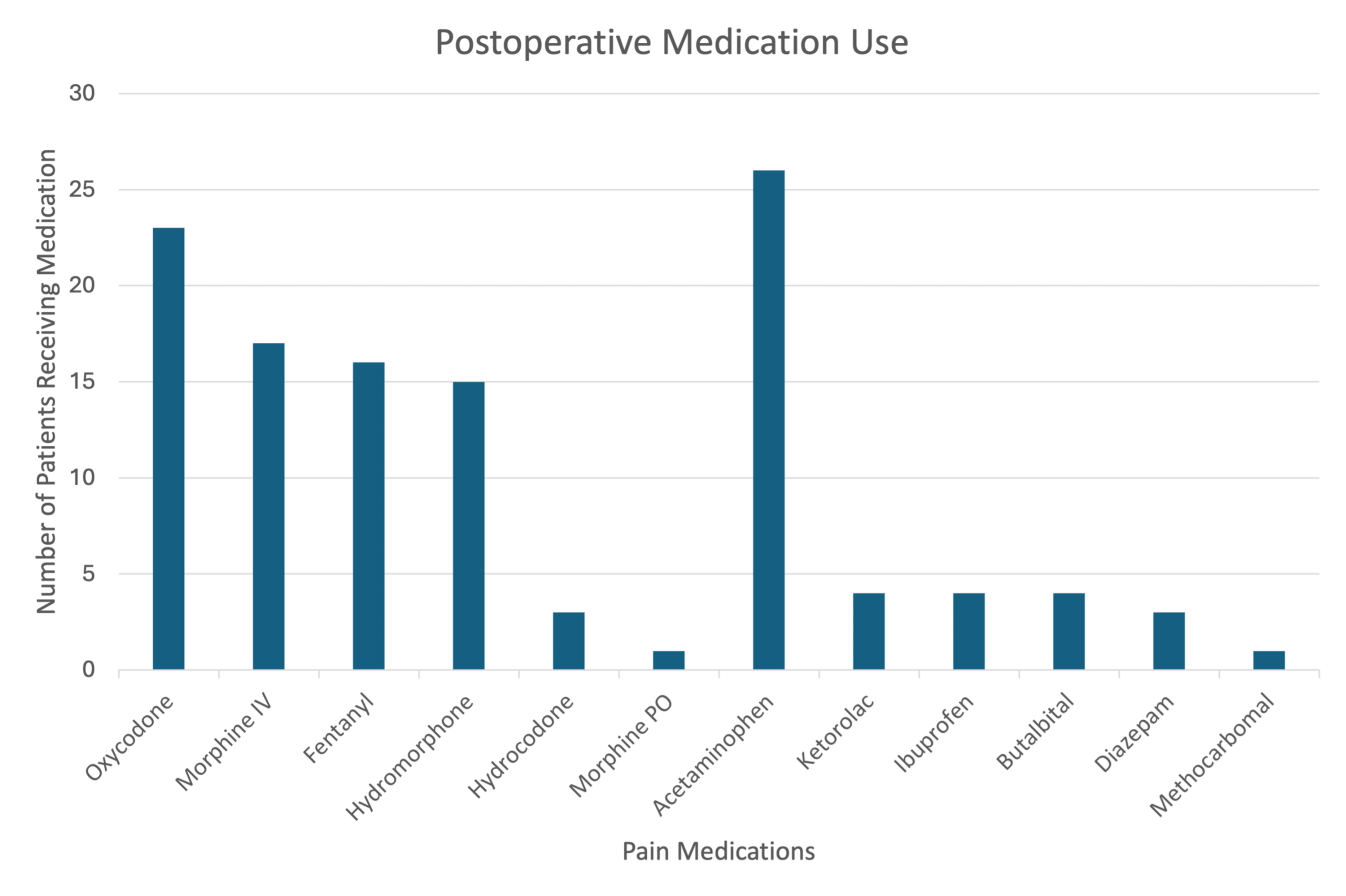
Distribution of Postoperative Multimodal Analgesic Use. The figure illustrates the frequency with which opioids, NSAIDs, acetaminophen, and adjunctive medications were prescribed postoperatively. While all patients received opioid analgesia, use of non-opioid modalities varied considerably. Oxycodone and acetaminophen were the most universally used medications. IV: intravenous; NSAIDs: non-steroidal anti-inflammatory drugs; PO: by mouth

Postoperative analgesic regimens were heterogeneous, and no standardized MMA protocol was identified across participating centers. Although most patients received opioid analgesia with at least one non-opioid adjunct, the specific agents, timing, and duration of therapy varied substantially. Accordingly, medication use was summarized descriptively rather than analyzed as a uniform treatment pathway. All patients received opioid analgesia during their inpatient course. Non-opioid adjuncts were used inconsistently and included acetaminophen (n = 26), NSAIDs (n = 7), butalbital (n = 4), diazepam (n = 3), and methocarbamol (n = 1). Specific drug classes and medications are described in Table 2. On average, patients received 3.89 unique pain medications (range, 1-7). The distribution of postoperative analgesic use is shown in Figure 1. The average MME per patient during the initial hospital stay was 33,015.13 (range, 22.5 to 360,126).

**Table 2:** Postoperative Pain Medications Utilized Following Surgical Intervention for Chiari Malformation. IV: intravenous; NSAIDs: non-steroidal anti-inflammatory drugs; PO: by mouth

Drug Class	Specific Medications
Opioids	Fentanyl (IV), hydromorphone (PO), hydromorphone (IV), hydrocodone (PO), oxycodone (PO), morphine (IV), morphine (PO)
NSAIDs	Ibuprofen (PO), ketorolac (IV)
Barbiturates	Butalbital (PO)
Benzodiazepines	Diazepam (PO)
Miscellaneous	Acetaminophen (PO), ketamine (IV), methocarbamol (PO)

Despite heterogeneous multimodal analgesic use during hospitalization, a substantial proportion of patients returned to the ED following discharge, most commonly for headache or cranial pressure symptoms. Eleven patients (40.7%) presented to the ED during the postoperative period, frequently requiring additional pharmacologic intervention, including opioids, butalbital-containing medications, ketorolac, and corticosteroids. ED presentations were commonly associated with further diagnostic evaluation, with 63.6% undergoing computed tomography (CT) and 18.2% undergoing magnetic resonance imaging (MRI). Two patients (18.2% of ED presentations; 7.4% of the total cohort) were subsequently found to have cerebrospinal fluid (CSF) leaks requiring operative intervention.

Inpatient clinical course was variable but generally consistent with standard postoperative recovery for cranial surgery. Patients required intravenous analgesia for an average of two days (range, 0-5) prior to transition to oral medications. The mean hospital LOS was 3.25 days (range, 1-9). The average number of postoperative imaging studies obtained during the index hospitalization was 1.04 per patient (range, 0-3). At discharge, 22 patients were prescribed oral opioid medications.

Exploratory analysis demonstrated that NSAID use was associated with lower average pain scores on postoperative day 0 (p = 0.025). No clear trends were identified between specific analgesic regimens and opioid consumption, LOS, or post-discharge healthcare utilization. Given the heterogeneity of treatment strategies and limited sample size, further comparative analysis was not pursued.

## Discussion

This study demonstrates that postoperative pain following Chiari malformation decompression represents a significant and underrecognized clinical challenge with meaningful downstream consequences. Despite the use of multimodal analgesic strategies during hospitalization, over 40% of patients in this cohort returned to the ED for pain-related complaints after discharge. These encounters frequently required additional pharmacologic intervention. The high rate of postoperative ED utilization observed in our cohort is consistent with prior literature. A previous study evaluating ED visits following suboccipital decompression for Chiari I malformation reported that approximately 25% of patients presented to the ED within 30 days of surgery, most commonly for pain-related complaints such as headache [21]. The even higher rate observed in our cohort underscores the persistent and often refractory nature of postoperative pain in this population and highlights the substantial burden it places on both patients and healthcare systems. Collectively, these findings suggest that postoperative pain following Chiari decompression remains a significant clinical challenge despite heterogeneous analgesic strategies.

Postoperative pain in Chiari patients appears to differ from that observed in other neurosurgical populations. The occipital and cervicogenic pain associated with posterior fossa decompression may be more severe, prolonged, and less responsive to conventional analgesic regimens. Rather than following a predictable postoperative trajectory, pain in this population may behave more like a persistent pain syndrome, contributing to recurrent healthcare utilization after discharge. The high rate of ED visits observed in this study supports prior reports demonstrating frequent post-discharge presentations driven by refractory headache and pressure-related symptoms [20,21]. Importantly, these visits often result in additional opioid exposure and repeat imaging, which may not alter clinical management but nonetheless contribute to patient burden and healthcare costs.

The variability in postoperative analgesic regimens observed across this cohort further highlights the absence of standardized, procedure-specific pain management pathways for Chiari decompression. Patients received a wide range of medication combinations, with inconsistent incorporation of non-opioid adjuncts. Although NSAID use was associated with improved pain scores on postoperative day 0, this benefit did not persist, and no clear reduction in opioid requirements or LOS was observed. Rather than suggesting ineffectiveness, these findings likely reflect variability in dosing, timing, and patient selection, as well as the limited power of this study to detect modest effects. Importantly, these results underscore that isolated pharmacologic interventions are unlikely to address the broader issue of persistent postoperative pain in this population without a more structured and standardized approach.

From a healthcare systems perspective, the high rate of post-discharge ED utilization represents a substantial and potentially modifiable burden. Each ED visit entails significant resource utilization, including staffing, pharmacologic management, and often advanced imaging. National estimates suggest that the mean charge per ED visit increased from $2,061 in 2010 to $3,516 in 2016, highlighting the considerable financial impact of unplanned care [23]. In addition to financial impacts, there are also indirect costs borne by patients such as time, anxiety, and disruption of recovery. The frequent use of CT and MRI in this cohort also raises concerns regarding unnecessary radiation exposure and healthcare expenditures. These findings highlight substantial postoperative healthcare utilization following Chiari decompression and suggest opportunities for further study of postoperative care pathways and outpatient symptom management.

An important implication of these findings is the need to reassess both patient selection and perioperative expectation setting in individuals undergoing Chiari decompression. The high proportion of patients returning to the ED with uncontrolled pain suggests that postoperative symptoms may not be fully anticipated or adequately contextualized during preoperative counseling. Prior literature has demonstrated that preoperative education and expectation setting can improve postoperative pain outcomes and patient experience, and may reduce unplanned healthcare utilization [24]. In addition to optimizing analgesic regimens, surgeons must ensure that patients have a clear understanding of the expected severity and duration of postoperative pain, particularly given the unique and often refractory nature of occipital and cervicogenic symptoms following posterior fossa surgery.

Taken together, these findings highlight substantial heterogeneity in postoperative analgesic practices and persistent healthcare utilization following Chiari decompression. Future investigation into more standardized perioperative pain management pathways, multimodal analgesic strategies, and postoperative follow-up approaches may help better characterize factors associated with postoperative recovery and healthcare utilization in this patient population. Additionally, incorporation of ERAS-informed principles tailored to neurosurgical patients may represent a potential area for future study.

Limitations

This study has several limitations. Its retrospective design introduces the potential for incomplete documentation and variability in recorded pain scores, medication administration, and postoperative follow-up. The relatively small sample size limits statistical power and precludes definitive conclusions regarding the effectiveness of specific analgesic regimens. Additionally, the substantial heterogeneity of treatment approaches reflects real-world clinical practice but limits the ability to perform robust comparative analyses.

Although all patients underwent Chiari-related decompressive procedures, the cohort included multiple operative approaches, including posterior fossa decompression, occipital craniectomy, and decompression with C1 laminectomy, as well as patients with both Chiari I and Chiari II malformations. Surgical techniques also varied with respect to intradural versus extradural decompression, which may influence postoperative pain burden, recovery trajectory, CSF-related complications, and healthcare utilization. These procedural differences likely contributed to variability in postoperative outcomes and pain experiences across the cohort. Accordingly, the findings should be interpreted as a descriptive assessment of postoperative pain and healthcare utilization across real-world Chiari decompression practice rather than as procedure-specific or diagnosis-specific estimates.

The cohort additionally demonstrated a high prevalence of preexisting headache disorders, elevated BMI, chronic pain syndromes, and psychiatric comorbidities, all of which may independently contribute to postoperative pain burden and healthcare utilization irrespective of analgesic regimen. These baseline characteristics represent important potential confounders and limit causal interpretation regarding postoperative pain management strategies. Furthermore, two postoperative ED presentations were ultimately attributable to CSF leak requiring reoperation rather than isolated pain-related complaints, which may partially inflate estimates of pain-related healthcare utilization.

Selection and surveillance bias should also be considered. Exclusion of patients with insufficient operative or postoperative documentation may preferentially exclude patients with uncomplicated postoperative courses and therefore introduce selection bias. Additionally, postoperative ED presentations were identified through participating health system electronic medical records, and out-of-network ED visits may therefore have been underdetected, potentially underestimating total postoperative healthcare utilization.

Finally, detailed characterization of preoperative opioid exposure, chronic outpatient analgesic use, opioid refill patterns, and duration of postoperative opioid therapy was not consistently available across institutions and therefore could not be reliably analyzed, despite their likely association with postoperative pain trajectories and healthcare utilization. The study also did not directly quantify the financial cost associated with ED visits and imaging, although these events are recognized contributors to increased healthcare expenditure.

Collectively, these limitations primarily affect interpretability and generalizability rather than simply statistical precision and should be considered when evaluating the findings of this exploratory retrospective study. Despite these limitations, this study provides important descriptive data highlighting the postoperative pain burden and associated healthcare utilization experienced by patients undergoing Chiari decompression.

Future directions

Future research should focus on prospective evaluation of standardized multimodal pain protocols tailored to Chiari decompression, with particular attention to reducing post-discharge pain burden and associated healthcare utilization. Studies incorporating cost analysis and patient-reported outcomes will be critical to further define the impact of these interventions. By shifting focus from isolated pharmacologic comparisons to comprehensive perioperative care pathways, there is an opportunity to meaningfully improve both patient experience and system-level outcomes in this challenging population.

## Conclusions

Postoperative pain and healthcare utilization following Chiari malformation decompression represent important clinical challenges. In this exploratory retrospective cohort, postoperative analgesic strategies were heterogeneous, and a substantial proportion of patients required postoperative ED evaluation, frequently associated with additional pharmacologic intervention and diagnostic imaging. These findings highlight the persistent postoperative symptom burden experienced by this patient population and may help inform future prospective evaluation of perioperative pain management pathways and postoperative care strategies following Chiari decompression.
